# Berberine Alleviates 1-Methyl-3-Nitro-1-Nitrosoguanidine-Induced Chronic Atrophic Gastritis in Rats

**DOI:** 10.5152/tjg.2025.24065

**Published:** 2025-04-07

**Authors:** Lingling Wang, Liqun Xie

**Affiliations:** 1 Nanjing Medical University, Jiangsu, China; 2 The First Affiliated Hospital with Nanjing Medical University, Jiangsu, China

**Keywords:** Berberine, chronic atrophic gastritis, intestinal flora, MAPK, NF-κB

## Abstract

**Background/Aims:**

Berberine (BBR), an isoquinoline alkaloid derived from *Berberis *plants, exhibits anti-inflammatory, anti-cancer, and antioxidant properties. This study explored the role of BBR in chronic atrophic gastritis (CAG).

**Materials and Methods:**

The 1-methyl-3-nitro-1-nitrosoguanidine and an irregular diet were used to establish the CAG model. Chronic atrophic gastritis rats were administered BBR at different doses via gavage, and teprenone (TEP) served as the positive control drug. We monitored and measured changes in body weight and food intake, pepsin activity, and gastric acid levels in the rats. Hematoxylin and eosin staining was utilized to scan the pathological condition in the gastric mucosal tissue of rats, while enzyme-linked immunosorbent assay was utilized to analyze alterations in serum inflammatory factors and hormone levels. Western blot was employed to evaluate protein expression. Additionally, 16S rRNA was conducted to assess changes in the intestinal flora of CAG rats.

**Results:**

Berberine increased body weight and food intake, improved gastric atrophy, and enhanced pepsin activity and total acidity of gastric juice in CAG rats. BBR treatment led to decreased levels of inflammation factors and motilin, while gastrin and somatostatin levels were elevated in CAG rats. Additionally, BBR inhibited the NF-κB and MAPK pathway in these rats. Berberine treatment also regulated the composition and abundance of intestinal flora. These microbiome alterations suggest a possible role in modulating gut inflammation associated with CAG.

**Conclusion:**

Berberine may alleviate CAG injury by reducing inflammation and regulating intestinal flora, which may be closely associated with the NF-κB and MAPK pathways.

**Main Points**

- Berberine alleviates the symptoms in chronic atrophic gastritis (CAG) rats.
- Berberine inhibits inflammation and enhances the secretion of gastrointestinal hormones in CAG rats.
- Berberine regulates the NF-κB and MAPK pathways in CAG rats.
- Berberine affects the changes of intestinal flora in CAG rats.

## Introduction

Chronic atrophic gastritis (CAG) is a common chronic digestive disorder characterized by the atrophy of gastric glands, thinning of the gastric mucosa, and thickening of the mucosal basal layer.^1^ It is widely recognized that CAG is considered a precancerous lesion for gastric cancer (GC).^2^ Persistent inflammation is an important factor in CAG pathogenesis,^3^^,^^4^ and it is also linked to an elevated risk of GC.^5^^,6^ Consequently, effective treatment of CAG is crucial for preventing and reducing the incidence of GC.

Berberine (BBR) is an isoquinoline alkaloid found in various *Berberis* species, including *Berberis aquifolium*, *Berberis aristata*, and *Phellodendron amurense*.^7^ Studies have demonstrated that BBR exhibits anti-cancer effects against GC.^8^ Berberine has been shown to inhibit the activation of innate immune cells and promote colonic epithelial repair in a colitis model.^9^ Additionally, accumulating evidence suggests that the intestinal flora is a target for the multifunctional effects of BBR.^10^ The study by Liu et al^11^ has proved the effect of BBR on the inhibition of *Helicobacter pylori* infection, mucosal inflammation, and the promotion of ulcer healing. Furthermore, BBR was demonstrated to have a protective effect on gastric injury in CAG rats.^12^ The regulatory role of BBR on inflammatory pathways was also found in various diseases.^13^^-^^16^ However, whether BBR can affect CAG by regulating intestinal flora and inflammatory pathways still needs to be further studied.

1-Methyl-3-nitro-1-nitrosoguanidine (MNNG) is a chemical carcinogen that can induce cell cycle arrest, DNA damage, and cell death.^17^ 1-Methyl-3-nitro-1-nitrosoguanidine mimics the conversion of nitrate in the stomach into carcinogenic substances such as nitrite, which can lead to CAG.^18^ The irregular diet simulates dietary risk factors associated with CAG in humans, enhancing the model’s clinical relevance.^19^^,^^20^ Consequently, MNNG is widely used in the development of CAG modeling. Thus, we constructed a CAG rat model utilizing MNNG and an irregular diet to explore the role of BBR in CAG and its probable mechanisms, with the aim of providing a solid foundation for CAG treatment.

## Materials and Methods

### Chronic Atrophic Gastritis Modeling

The rats (7-8 weeks, male, Vital River Laboratories) were adaptively housed for 1 week prior to modeling under specific pathogen-free settings, with 25°C, 55% relative humidity, and a cycle of 12 hours light: 12 hours dark. For CAG modeling, the rats were administered MNNG (60397ES08, Yeasen Biotechnology (Shanghai) Co., Ltd) at 170 μg/mL in a light-tight bottle. The MNNG solution was replaced daily. Meanwhile, the rats received a mixture of 30% ethanol and 2% sodium salicylate by gavage (1 mL/100 g) 3 times a week, and they were fasted for 1 hour before and after each gavage. Additionally, rats were provided with an irregular diet, alternating between 1 full day and 1 fast day, for 12 weeks. Subsequently, the CAG rats were randomly assigned to groups: the CAG (no treatment), CAG+BBR-L (14 mg/kg/day BBR, 54063ES25, Yeasen Biotechnology), CAG+BBR-H (28 mg/kg/day BBR), and CAG+teprenone (TEP) (18.75 mg/kg/day TEP as a positive control, S86193, Yuanye, Shanghai, China). Body weight and daily food intake were recorded. After 4 weeks of treatment with BBR or TEP, blood was collected before euthanasia. The gastric mucosal tissue and rat colon feces were collected for subsequent research. The study was authorized by the Experimental Animal Ethics Committee of Nanjing Medical University (approval number: 2309048, date: September 12, 2023).

### Hematoxylin and Eosin Staining

The tissue was fixed in 10% formaldehyde, dehydrated using gradient alcohol, vitrified with xylene, embedded in paraffin, and then sectioned into 5 μm-thick slices. The slices were dewaxed, rehydrated, and stained with hematoxylin for 20 minutes, followed by counterstaining with eosin for 3 minutes. The histopathology was observed using a microscope.

### Detection of Gastric Juice

The gastric juice was centrifuged at 2400 × *g* to obtain the supernatant, which was measured as total gastric juice (mL). A 1% phenolphthalein solution was added as an indicator to 1 mL of gastric juice, and 0.1 mol/L NaOH was titrated until a light pink color appeared. Total acidity (mmol/L) was calculated utilizing the formula: Total acidity = (NaOH volume × NaOH normality × 100) / 0.1.

### Enzyme-Linked Immunosorbent Assay

The level of pepsin (BC2320, Solarbio, Beijing, China) in gastric juice, and serum levels of IL-6 (E-EL-R0015, Elabscience, Wuhan, China), TNF-α (E-EL-R2856, Elabscience), IL-10 (CSB-E04595r, CUSABIO, Wuhan, China), gastrin (CSB-E12743r, CUSABIO), motilin (CSB-E08208r, CUSABIO), somatostatin (CSB-E08204r, CUSABIO) were detected by enzyme-linked immunosorbent assay (ELISA) kits

### Western Blot

RIPA Lysis Buffer (P0013, Beyotime) was employed to extract total protein, and BCA Protein Assay Kit (ab102536, Abcam) was employed to evaluate the protein concentration. The protein expression was assessed according to the provided instructions. The bands were visualized using the ECL Substrate Kit (ab133406, Abcam) and analyzed with ImageJ software. The primary antibodies utilized in this study included p-p65 (80379-2-RR, Proteintech Group, Inc), p65 (10745-1-AP, Proteintech), p-IkBα (ab133462, Abcam), IkBα (ab32518, Abcam), p-p38 (28796-1-AP, Proteintech), p38 (14064-1-AP, Proteintech), p-ERK (28733-1-AP, Proteintech), ERK (11257-1-AP, Proteintech), p-JNK (9251, Cell Signaling Technology), JNK (9252, Cell Signaling Technology), and GAPDH (10494-1-AP, Proteintech). Goat Anti-Rabbit IgG H&L (HRP) (ab6721, Abcam) was the secondary antibody.

### Fecal Microbiota Analysis

The fecal samples were collected for 16S rDNA sequencing. The QIAamp PowerFecal Pro DNA Kit (51804, Qiagen) was utilized for total DNA extraction. The DNA was quantified using the Nanodrop method, and 1.2% agarose gel electrophoresis was utilized to assess DNA extraction quality. Following amplification, the Quant-iT PicoGreen dsDNA Assay Kit (P11496, Thermo Fisher Scientific) with a microplate reader (BioTek, FLx800) was utilized to quantify the PCR products. Each sample was mixed in equal amounts to form a template, and the library index and splicing sequence required for Illumina sequencing were added to the template for a second PCR. After library purification, the High Sensitivity DNA Kit (5067-4626, Agilent) was utilized for sample testing and accurate quantification with a bioanalyzer (Agilent). The libraries were subjected to 250 bp paired-end sequencing on a MiSeq System (Illumina) according to the standard procedure.

### Bioinformatics

The targets of BBR were obtained from the CTD (https://ctdbase.org/). Kyoto Encyclopedia of Genes and Genomes (KEGG) enrichment analysis of BBR-regulated genes was performed utilizing DAVID (https://david.ncifcrf.gov/).

### Statistical Analysis

The experiment results were analyzed using GraphPad Prism 9.0 (GraphPad Software, Boston, Massachusetts, USA). All measurements were repeated at least 3 times. Data are presented as mean ± standard deviation. Differences between groups were analyzed using one-way ANOVA and Tukey’s test. *P* < .05 indicates statistical significance.

## Results

### BBR Alleviates the Symptoms in Chronic Atrophic Gastritis Rats

**Figure 1.**
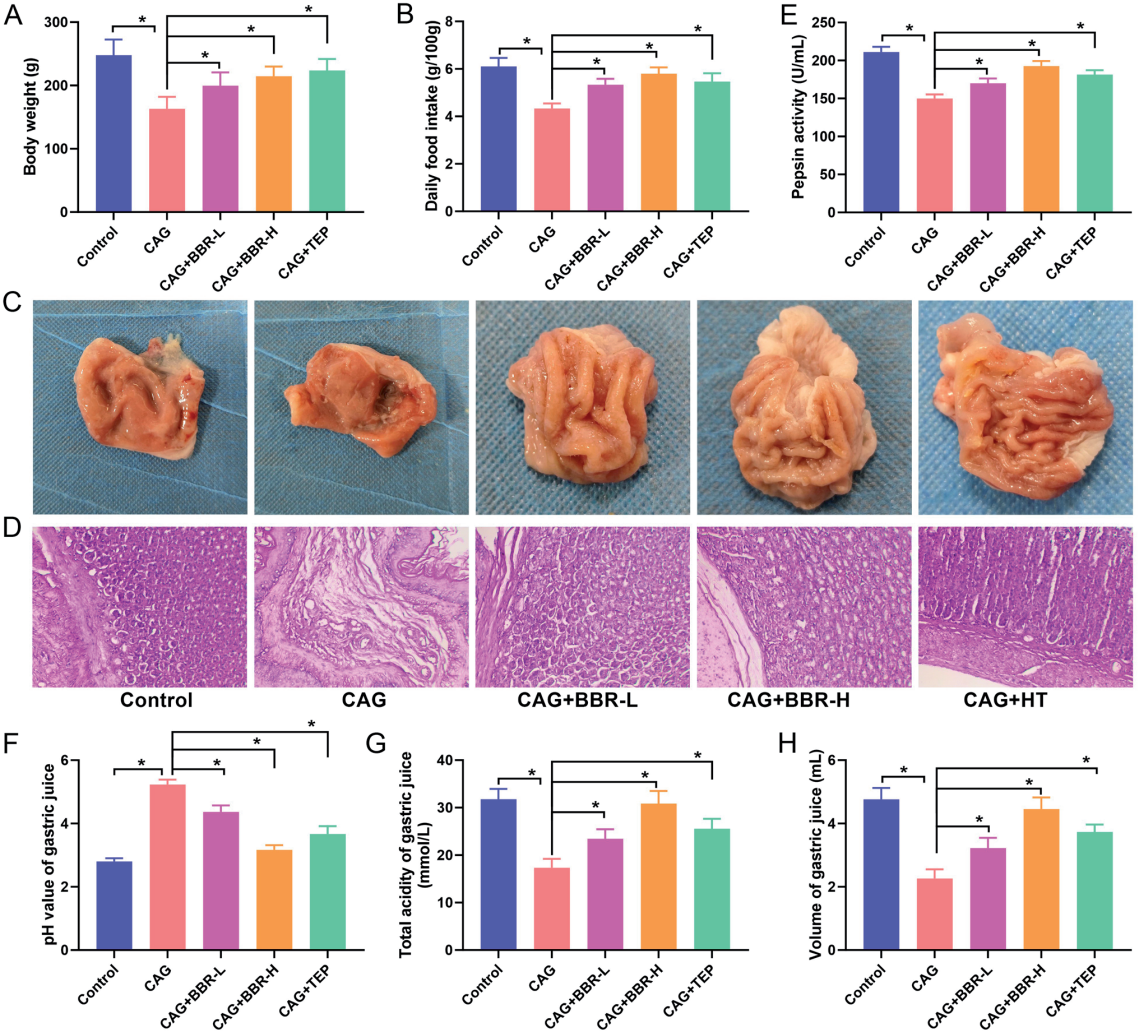
BBR alleviates the symptoms of CAG rats. (A) The body weight of rats. (B) The daily food intake of rats. (C) The macroscopic view of the gastric mucosa. (D) The pathological changes of the gastric mucosa. (E) The pepsin activity of rats. (F) The pH value of gastric juice. (G) The total acidity of gastric juice. (H) The volume of gastric juice. *P < .05.

The body hair of control rats is smooth, dense, and glossy, indicating a good mental state and stable mood during weighing and other procedures. Their stool is yellowish-brown in color. In contrast, the body hair of the CAG group is withered, sparse, and dull, with a tendency to fall out. The mental state of these CAG rats is flagging, and they exhibit emotional fluctuations, as well as behaviors such as biting, tearing, and grasping during weighing and other operations. Some rats in this group show signs of perianal contamination and loose stools. At the 12th week, the gastric mucosa in the CAG group exhibited a reduction in intrinsic glands, confirming the success of the model. Following the establishment of the CAG model, the effects of BBR on CAG were evaluated. The CAG rats demonstrated decreased body weight and food intake (Figure 1A and B), which were significantly increased by BBR and the positive control drug TEP. As illustrated in Figure 1C, the gastric tissue in the CAG group appeared slightly pale in comparison with the control group, with fewer gastric folds. Both BBR and TEP improved gastric morphology (Figure 1C). The CAG model group exhibited evident gastric atrophy and inflammation. Remarkably, both gastric gland atrophy and inflammation were ameliorated following BBR treatment (Figure 1D). Similar results to those observed in BBR-treated rats were also noted in the TEP group (Figure 1D). We assessed the effects of BBR treatment on gastric secretory function. In comparison with the control group, the pepsin activity, acidity, and total volume of gastric juice were lower, while the pH of gastric juice was higher in the CAG group. Notably, pepsin activity, acidity, and total volume of gastric juice increased while pH decreased after BBR and TEP treatment (Figure 1E-H).

### Berberine Inhibits Inflammation and Enhances the Secretion of Gastrointestinal Hormones in Chronic Atrophic Gastritis Rats

**Figure 2.**
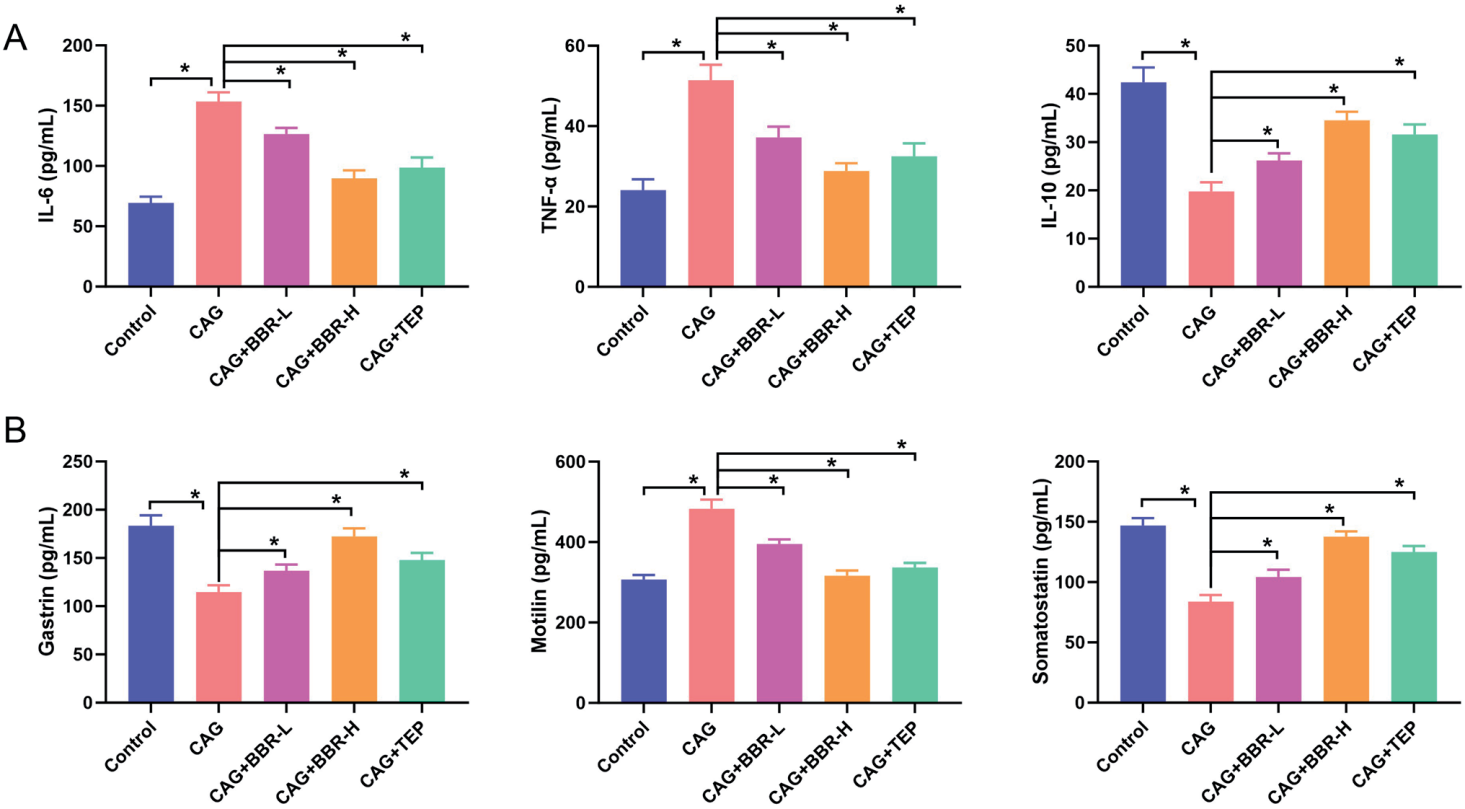
BBR inhibits inflammation and improves the secretion of gastrointestinal hormones in CAG rats. (A) The serum levels of IL-6, TNF-α, and IL-10 in CAG rats. (B) The serum levels of gastrin, motilin, and somatostatin in CAG rats. *P < .05.

Chronic atrophic gastritis linked with changes in inflammatory factors and gastrointestinal hormones.^21^ The serum levels of inflammation factors, as well as gastric mucosal hormones in CAG rats, were measured using ELISA. The levels of IL-6 and TNF-α were elevated, while the level of IL-10 was reduced in the CAG group (Figure 2A). Berberine and TEP eliminated the effect of CAG modeling on the inflammatory response. Additionally, serum levels of gastrin and somatostatin were decreased, while the motilin level was increased in CAG rats (Figure 2B). Berberine and TEP treatment elevated the serum levels of gastrin and somatostatin and reduced the level of motilin in the CAG rats.

### Berberine Regulates the NF-κB Signaling Pathway and MAPK Signaling Pathway in Chronic Atrophic Gastritis Rats

**Figure 3.**
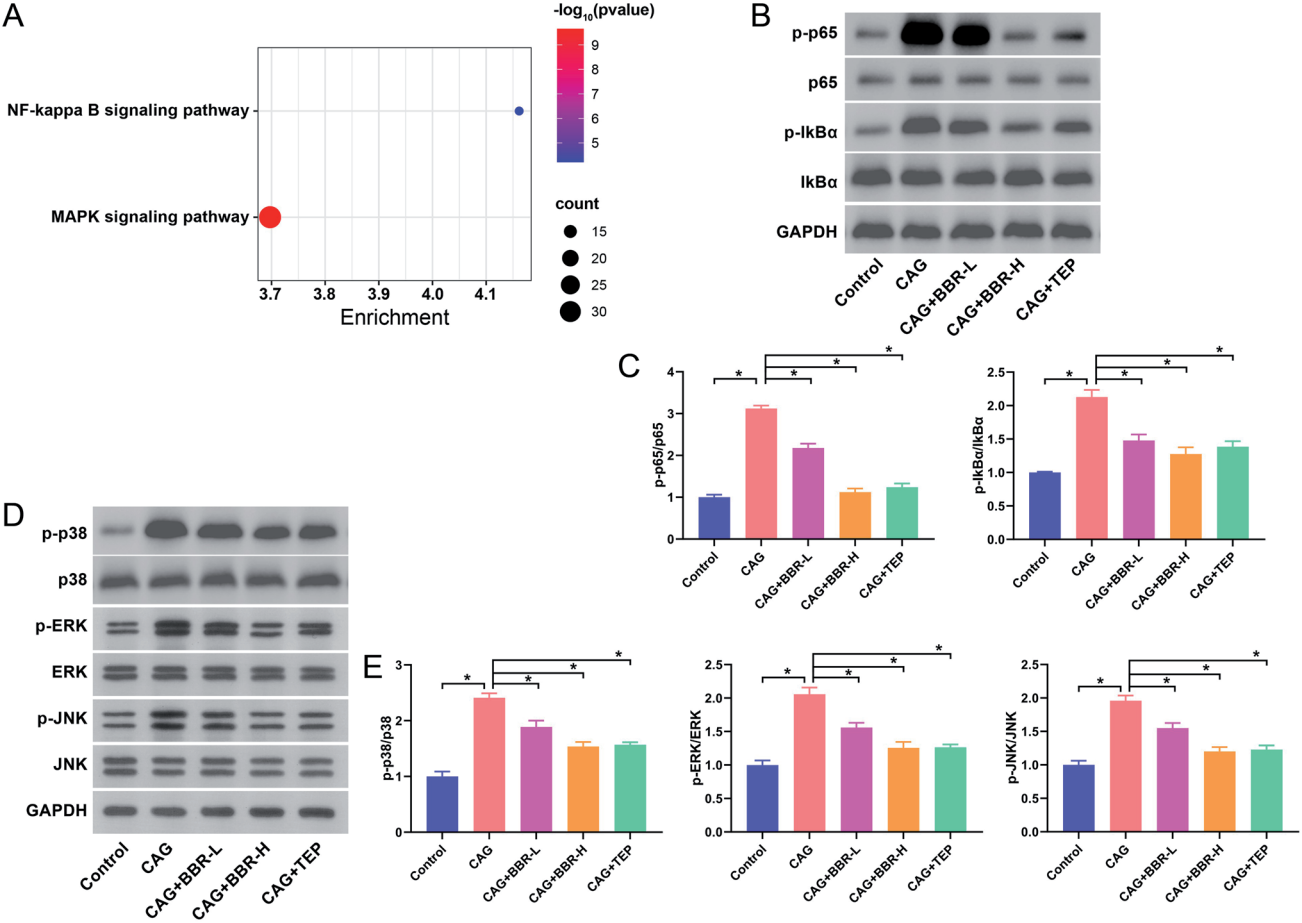
BBR regulates NF-κB and MAPK signaling pathways in CAG rats. (A) Bubble chart of KEGG analysis. (B) The protein expression of p-IκBα/IκBα and p-p65/p65 in gastric mucosa tissues. (C) The relative expression of p-IκBα/IκBα and p-p65/p65 in gastric mucosa tissues. (D) The protein expression of p-p38/p38, p-ERK/ERK, and p-JNK/JNK in gastric mucosa tissues. (E) The relative expression of p-p38/p38, p-ERK/ERK, and p-JNK/JNK in gastric mucosa tissues. *P < .05.

We downloaded the targets of BBR from CTD and performed KEGG enrichment analysis. The results indicated that BBR regulates both the NF-κB and MAPK pathways (Figure 3A). We then examined the effects of BBR treatment on these signaling pathways in CAG rats (Figure 3B-E). The levels of p-IκBα/IκBα and p-p65/p65 (Figure 3B and C), p-ERK/ERK, p-p38/p38, and p-JNK/JNK (Figure 3D-E) were markedly enhanced in CAG rats. BBR and TEP suppressed the levels of these proteins that were elevated due to CAG modeling.

### Berberine Affects the Changes of Intestinal Flora in Chronic Atrophic Gastritis Rats

**Figure 4.**
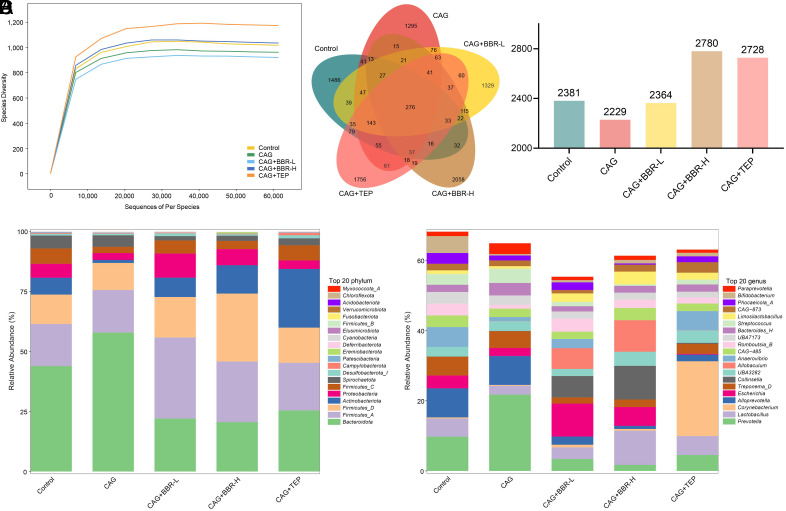
BBR affects the changes of intestinal flora in CAG rats. (A) The species rarefaction curves of intestinal flora. (B) Venn plot of the composition of rat intestinal flora. (C) The composition of bacterial community of each group at the phylum level. (D) The composition of bacterial community of each group at the genus level.

The fecal samples from the rats were subsequently analyzed to assess the diversity of microbial communities. The operational taxonomic units (OTUs) of each group did not increase with additional sequencing (Figure 4A). The composition of the rat intestinal flora is illustrated in the Venn diagram and bar chart in Figure 4B. Chronic atrophic gastritis modeling resulted in a decrease in the intestinal flora of the rats. Conversely, BBR and TEP treatments increased the intestinal flora diversity in CAG rats, particularly with BBR-H and TEP. Figure 4C and D present the species composition analysis of the identified intestinal flora in the samples from each group. In terms of phylum-level analysis results (Figure 4C), the abundance of *Actinobacteriota*, *Proteobacteria*, and *Firmicutes*_*C *were reduced in the CAG group in comparison with the control group, while the abundance of *Bacteroidota *was raised. Compared to the CAG group, the abundance of *Firmicutes*_*A*,*Firmicutes_D*,* Actinobacteria*,and *Proteobacteria *was increased in the BBR treatment group, whereas the abundance of *Bacteroidota *and *Spirochaetota *was decreased. Compared to the CAG group, the abundance of *Actinobacteriota *and *Firmicutes*_*A*, *Firmicutes_C*,and* Campylobacterota* was raised in the TEP group, but the abundance of *Bacteroidota* and *Spirochaetota* was decreased. In the analysis at the genus level (Figure 4D), the abundance of *Lactobacillus*, *Escherichia*, *Anaerovibrio*, *Romboutsia_B*, and *Bifidobacterium* was decreased in the CAG group, while the abundance of *Prevotella*, *Bacteroides_H*, and *Paraprevotella *was increased. Compared to the CAG group, the abundance of *Prevotella*, *Alloprevotella*, *Bacteroides*_*H*, *Streptococcus*, and *Paraprevotella* was decreased in the BBR groups, while the abundance of *Lactobacillus*, *Escherichia*, *Collinsella*, *Allobaculum*, *Romboutsia_B*, and *Limosilactobacillus* was raised. In the TEP group, the abundance of *Prevotella*, *Alloprevotella*, *Escherichia*, *Streptococcus*,* and Paraprevotella* was decreased, while the abundance of *Lactobacillus*, *Corynebacterium*, *Anaerovibrio*, *Limosilactobacillus*, and *CAG*-*873 *was increased.

## Discussion

Chronic atrophic gastritis is a prevalent digestive system disorder. It is considered to be a precancerous lesion for GC and carries a high risk of progression to malignancy.^2^ Berberine has demonstrated significant therapeutic effects on various diseases. Research indicates that BBR effectively treats ulcerative colitis induced by dextran sulfate sodium via the inhibition of the IFN-γ signaling pathway.^22^ Furthermore, BBR may ameliorate gastric tissue damage in CAG rats by modulating the TGF-β1/PI3K signaling pathway.^12^ Our findings reveal that BBR regulates the NF-κB and MAPK pathways and alters the abundance of intestinal flora in CAG rats. This suggests that these may underlie the therapeutic effects of BBR in CAG.

As CAG progresses, gastric acid secretion diminishes, and pepsin activity decreases.^23^ Treatment with BBR results in increased body weight and food intake in CAG rats, improved gastric mucosal injury, enhanced gastric acid secretion, and elevated pepsin activity. These findings suggest that BBR therapy alleviates symptoms associated with MNNG-induced CAG. Gastrin is a hormone produced by G cells in the gastric antrum that stimulates gastric acid secretion and plays both nutritional and protective roles in the gastric mucosa.^24^ Motilin enhances gastrointestinal tract motility and contributes to gastric emptying and food digestion.^25^ Somatostatin, primarily produced by mucosal endocrine cells, has somatostatin analogues that are promising drugs for gastrointestinal tumors treatment.^26^ We found that BBR treatment promoted the secretion of gastrin and somatostatin while inhibiting motilin secretion. Persistent inflammatory reactions in the gastric mucosa are critical factors affecting the progression of CAG.^27^ Pro-inflammatory cytokines play a vital role in CAG development.^28^^,^^29^ The gastric mucosa in CAG exhibits significant inflammatory infiltration.^30^ Berberine has been reported to alleviate non-alcoholic fatty liver disease by inhibiting the inflammatory response.^32^ By reducing inflammation, BBR can mitigate intestinal damage induced by LPS.^31^ A previous study confirmed that certain inflammatory cytokines were elevated in CAG rats.^21^ We found BBR inhibited inflammation response. These results suggest that BBR exhibited an inhibitory effect on inflammation and a beneficial regulatory effect on the secretion of intestinal hormones.

Activation of NF-κB leads to significant inflammation.^32^ Suppression of the NF-κB signaling pathway can alleviate MNNG-induced chronic gastric disorders.^32^ Inhibition of NF-κB signaling has been shown to alleviate the symptoms of CAG in mice.^33^ The MAPK pathway regulates cell proliferation, stress response, inflammation, and apoptosis.^34^ ERK and JNK are 2 subfamilies of the classical MAPK signaling pathway, activated by cellular stimuli such as inflammatory cytokines.^35^ Zhang et al^36^ demonstrated the inhibitory role of BBR on the MAPK signaling pathway in GC. In this study, we revealed that BBR modulated both the NF-κB and MAPK pathways. Berberine treatment reduced the levels of pathway-related proteins in gastric mucosa, indicating the inhibitory role of BBR on the NF-κB and MAPK signaling pathways. These results suggest that BBR alleviates CAG by inhibiting the NF-κB and MAPK pathways.

The intestinal flora consists of a diverse array of bacteria that play crucial roles in important metabolic functions and the regulation of gastrointestinal health.^4^ The abundance of microflora in patients with gastritis has changed significantly.^37^^,^^38^ Zhou et al^39^ demonstrated that modulating intestinal flora can inhibit the progression of CAG. This study identified intestinal dysbiosis in rats with CAG. Following treatment with BBR, there was a significant increase in the abundance of *Proteobacteria*, *Firmicutes*, *Actinobacteria*, *Lactobacillus*, *Escherichia*, *Collinsella*, *Allobaculum*, *Romboutsia_B*, and *Limosilactobacillus*, while the abundance of *Bacteroidota* and *Spirochaetota*, *Prevotella*, *Alloprevotella*, *Bacteroides_H*, *Streptococcus*, and *Paraprevotella* were decreased. These findings suggest that BBR may alleviate the symptoms of CAG by modulating the intestinal flora in rats.

Previous studies have reported that dysbiosis of the intestinal flora leads to changes in cytokine activity and activation of inflammatory signaling, including TLR4, TLR5, NF-κB, and MAPKs.^,^^40^ In this study, the NF-κB and MAPK pathways were overactivated in CAG rats, along with alterations in their intestinal flora. However, this study did not explore the association between gut microbiota regulation and the modulation of the NF-κB and MAPK signaling pathways, which represents a limitation of our research. Therefore, we plan to incorporate investigations in this area into our future research agenda.

Berberine can alleviate the symptoms of CAG by inhibiting the inflammatory response and enhancing the secretion of gastrointestinal hormones. The mechanism may be related to changes in the NF-κB pathway and MAPK pathway, and the relative abundance of intestinal flora.

## Data Availability

The datasets used and/or analyzed during the current study are available from the corresponding author upon reasonable request.

## References

[1] Park YH Kim N Review of atrophic gastritis and intestinal metaplasia as a premalignant lesion of gastric cancer. J Cancer Prev. 2015;20(1):25 40. (doi: 10.15430/JCP.2015.20.1.25) 25853101 PMC4384712

[2] Dinis-Ribeiro M Areia M de Vries AC Management of precancerous conditions and lesions in the stomach (MAPS): guideline from the European Society of Gastrointestinal Endoscopy (ESGE), European Helicobacter Study Group (EHSG), European Society of Pathology (ESP), and the Sociedade Portuguesa de Endoscopia Digestiva (SPED). Endoscopy. 2012;44(1):74 94. (doi: 10.1055/s-0031-1291491) 22198778 PMC3367502

[3] Chen P Cui Y Fu QY Lu YY Fang JY Chen XY. Positive relationship between p42.3 gene and inflammation in chronic non-atrophic gastritis. J Dig Dis. 2015;16(10):568 574. (doi: 10.1111/1751-2980.12282) 26316259

[4] Luo C Sun Z Li Z Zheng L Zhu X. Notoginsenoside R1 (NGR1) attenuates chronic atrophic gastritis in rats. Med Sci Monit. 2019;25:1177 1186. 30757999 10.12659/MSM.911512 PMC6381808

[5] Engstrand L Graham DY. Microbiome and gastric cancer. Dig Dis Sci. 2020;65(3):865 873. (doi: 10.1007/s10620-020-06101-z) 32040665 PMC8697197

[6] Jaroenlapnopparat A Bhatia K Coban S. Inflammation and gastric cancer. Diseases. 2022;10(3):35. (doi: 10.3390/diseases10030035) 35892729 PMC9326573

[7] Cicero AF Baggioni A. Berberine and its role in chronic disease. Adv Exp Med Biol. 2016;928:27 45. (doi: 10.1007/978-3-319-41334-1_2) 27671811

[8] Peng Z Wangmu T Li L Han G Huang D Yi P. Combination of berberine and low glucose inhibits gastric cancer through the PP2A/GSK3β/MCL-1 signaling pathway. Eur J Pharmacol. 2022;922:174918. (doi: 10.1016/j.ejphar.2022.174918) 35341784

[9] Luo Z Li Z Liang Z Berberine increases stromal production of Wnt molecules and activates Lgr5(+) stem cells to promote epithelial restitution in experimental colitis. BMC Biol. 2022;20(1):287. (doi: 10.1186/s12915-022-01492-z) PMC9759859 36528592

[10] Habtemariam S. Berberine pharmacology and the gut microbiota: a hidden therapeutic link. Pharmacol Res. 2020;155:104722. (doi: 10.1016/j.phrs.2020.104722) 32105754

[11] Liu Q Tang J Chen S Berberine for gastric cancer prevention and treatment: multi-step actions on the Correa’s cascade underlie its therapeutic effects. Pharmacol Res. 2022;184:106440. (doi: 10.1016/j.phrs.2022.106440) 36108874

[12] Tong Y Liu L Wang R Berberine attenuates chronic atrophic gastritis induced by MNNG and its potential mechanism. Front Pharmacol. 2021;12:644638. (doi: 10.3389/fphar.2021.644638) PMC8026873 33841162

[13] Haftcheshmeh SM Abedi M Mashayekhi K Berberine as a natural modulator of inflammatory signaling pathways in the immune system: focus on NF-κB, JAK/STAT, and MAPK signaling pathways. Phytother Res. 2022;36(3):1216 1230. (doi: 10.1002/ptr.7407) 35142403

[14] Zhao Y Tian X Liu G Wang K Xie Y Qiu Y. Berberine protects myocardial cells against anoxia-reoxygenation injury via p38 MAPK-mediated NF-κB signaling pathways. Exp Ther Med. 2019;17(1):230 236. (doi: 10.3892/etm.2018.6949) 30651787 PMC6307361

[15] Zhao FQ Zhao Y Liu JY Gou YJ Yang YQ. [ Effects of berberine on LPS /NF-κB and MAPK signaling pathways in PCOS model rats]. Zhongguo Ying Yong Sheng Li Xue Za Zhi Zhongguo Yingyong Shenglixue Zazhi Chin J Appl Physiol. 2022;38(2):181 186. (doi: 10.12047/j.cjap.6229.2022.029) 36031579

[16] Han Y Guo S Li Y Berberine ameliorate inflammation and apoptosis via modulating PI3K/AKT/NFκB and MAPK pathway on dry eye. Phytomedicine. 2023;121:155081. (doi: 10.1016/j.phymed.2023.155081) 37748390

[17] Gichner T Velemínský J. Genetic effects of N-methyl-N’-nitro-N-nitrosoguanidine and its homologs. Mutat Res. 1982;99(2):129 242. (doi: 10.1016/0165-1110(82)90057-4) 6750393

[18] Luo Y Liang M Yao W Functional role of lncRNA LOC101927497 in N-methyl-N’-nitro-N-nitrosoguanidine-induced malignantly transformed human gastric epithelial cells. Life Sci. 2018;193:93 103. (doi: 10.1016/j.lfs.2017.12.007) 29223541

[19] Balendra V Amoroso C Galassi B High-salt diet exacerbates H. pylori infection and increases gastric cancer risks. J Pers Med. 2023;13(9):1325. (doi: 10.3390/jpm13091325) 37763093 PMC10533117

[20] Sipponen P Maaroos HI. Chronic gastritis. Scand J Gastroenterol. 2015;50(6):657 667. (doi: 10.3109/00365521.2015.1019918) 25901896 PMC4673514

[21] Zhang J Wang H. Morroniside protects against chronic atrophic gastritis in rat via inhibiting inflammation and apoptosis. Am J Transl Res. 2019;11(9):6016 6023. 31632569 PMC6789209

[22] Kubistová V. [ Diagnosis of strabismus]. Cesk Oftalmol. 1987;43(1):9 11. 3581215

[23] Yang GT Zhao HY Kong Y Sun NN Dong AQ. Correlation between serum vitamin B12 level and peripheral neuropathy in atrophic gastritis. World J Gastroenterol. 2018;24(12):1343 1352. (doi: 10.3748/wjg.v24.i12.1343) 29599609 PMC5871829

[24] Rehfeld JF. Gastrin and the moderate hypergastrinemias. Int J Mol Sci. 2021;22(13):6977. (doi: 10.3390/ijms22136977) 34209478 PMC8269006

[25] Al-Missri MZ Jialal I StatPearls [Internet]. 2022 Sep 26.

[26] Curt AM Popa Ilie IR Cainap C Balacescu O Ghervan C. MicroRNAs and treatment with somatostatin analogs in gastro- entero-pancreatic neuroendocrine neoplasms: challenges in personalized medicine. J Gastrointestin Liver Dis. 2020;29(4):647 659. (doi: 10.15403/jgld-2866) 33331339

[27] Pimentel-Nunes P Libânio D Marcos-Pinto R Management of epithelial precancerous conditions and lesions in the stomach (MAPS II): European Society of Gastrointestinal Endoscopy (ESGE), European Helicobacter and Microbiota Study Group (EHMSG), European Society of Pathology (ESP), and Sociedade Portuguesa de Endoscopia Digestiva (SPED) guideline update 2019. Endoscopy. 2019;51(4):365 388. (doi: 10.1055/a-0859-1883) 30841008

[28] Chen J Wang W Zhang T Differential expression of phospholipase C epsilon 1 is associated with chronic atrophic gastritis and gastric cancer. PLoS One. 2012;7(10):e47563. (doi: 10.1371/journal.pone.0047563) PMC3471869 23077637

[29] Jeong M Park JM Han YM Dietary intervention of artemisia and green tea extracts to rejuvenate Helicobacter pylori-associated chronic atrophic gastritis and to prevent tumorigenesis. Helicobacter. 2016;21(1):40 59. (doi: 10.1111/hel.12229) 25864522

[30] Goldenring JR Mills JC. Cellular plasticity, reprogramming, and regeneration: metaplasia in the stomach and beyond. Gastroenterology. 2022;162(2):415 430. (doi: 10.1053/j.gastro.2021.10.036) 34728185 PMC8792220

[31] Izadparast F Riahi-Zajani B Yarmohammadi F Hayes AW Karimi G. Protective effect of berberine against LPS-induced injury in the intestine: a review. Cell Cycle (Georget Tex). 2022;21(22):2365 2378. (doi: 10.1080/15384101.2022.2100682) PMC9645259 35852392

[32] Barnabei L Laplantine E Mbongo W Rieux-Laucat F Weil R. NF-κB: at the borders of autoimmunity and inflammation. Front Immunol. 2021;12:716469. (doi: 10.3389/fimmu.2021.716469) PMC8381650 34434197

[33] Jiang JY Liu DJ Liu MX. The protective effect of NF-κB signaling pathway inhibitor PDTC on mice with chronic atrophic gastritis. Scand J Gastroenterol. 2021;56(10):1131 1139. (doi: 10.1080/00365521.2021.1953130) 34310252

[34] Gałgańska H Jarmuszkiewicz W Gałgański Ł. Carbon dioxide and MAPK signalling: towards therapy for inflammation. Cell Commun Signal. 2023;21(1):280. (doi: 10.1186/s12964-023-01306-x) PMC10566067 37817178

[35] Park JI. MAPK-ERK pathway. Int J Mol Sci. 2023;24(11):9666. (doi: 10.3390/ijms24119666) 37298618 PMC10253477

[36] Zhang Q Wang X Cao S Berberine represses human gastric cancer cell growth in vitro and in vivo by inducing cytostatic autophagy via inhibition of MAPK/mTOR/p70S6K and Akt signaling pathways. Biomed Pharmacother. 2020;128:110245. (doi: 10.1016/j.biopha.2020.110245) 32454290

[37] Park CH Lee AR Lee YR Eun CS Lee SK Han DS. Evaluation of gastric microbiome and metagenomic function in patients with intestinal metaplasia using 16S rRNA gene sequencing. Helicobacter. 2019;24(1):e12547. (doi: 10.1111/hel.12547) PMC6587566 30440093

[38] Zhang S Shi D Li M Li Y Wang X Li W. The relationship between gastric microbiota and gastric disease. Scand J Gastroenterol. 2019;54(4):391 396. (doi: 10.1080/00365521.2019.1591499) 30945954

[39] Zhou P Hao X Liu Y Determination of the protective effects of Hua-Zhuo-Jie-Du in chronic atrophic gastritis by regulating intestinal microbiota and metabolites: combination of liquid chromatograph mass spectrometer metabolic profiling and 16S rRNA gene sequencing. Chin Med. 2021;16(1):37. (doi: 10.1186/s13020-021-00445-y) PMC8088729 33933119

[40] Zhu HC Jia XK Fan Y Alisol B 23-acetate ameliorates azoxymethane/dextran sodium sulfate-induced male murine colitis-associated colorectal cancer via modulating the composition of gut microbiota and improving intestinal barrier. Front Cell Infect Microbiol. 2021;11:640225. (doi: 10.3389/fcimb.2021.640225) PMC8117151 33996624

